# Disgust memory enhancement extends to more accurate memory but not more false memories

**DOI:** 10.3758/s13421-024-01681-x

**Published:** 2025-01-20

**Authors:** Lucy A. Matson, Ella K. Moeck, Tyla R. Molyneux, Melanie K. T. Takarangi

**Affiliations:** 1 College of Education, Psychology and Social Work, Flinders University, GPO Box 2100, Adelaide, South Australia 5042 Australia; 2 School of Psychology, Adelaide University, Adelaide, South Australia Australia; 3 Melbourne School of Psychological Sciences, The University of Melbourne, Melbourne, Victoria Australia

**Keywords:** Disgust, Fear, Recognition, False memory, Posttraumatic stress

## Abstract

People show enhanced memory recall for disgust over fear, despite both being highly negative and arousing emotions. But does disgust’s ‘stickiness’ in memory result in more false memories for disgust versus fear? Existing research finds low false-memory rates for disgust *and* fear, perhaps from using image lures depicting content *unrelated* to target images. Therefore, we presented 111 participants with disgust, fear, (and neutral) images during an attention-monitoring task. After 24–48 hours, participants completed a recognition test, where they viewed ‘old’ (previously seen) and ‘new’ images (both *related* and *unrelated* lures) and indicated whether each image was ‘old’ or ‘new’. Relative to fear, participants experienced fewer false memories of disgust for *unrelated* lures, but similar false memories for *related* lures. Furthermore, participants’ attention was captured more by disgust than fear images, and correct recognition and memory sensitivity were enhanced for disgust relative to fear. Our findings suggest disgust memory enhancement extends to *accurate* memory, which has clinical implications.

**Supplementary information:**

The online version contains supplementary material available at 10.3758/s13421-024-01681-x.

Disgust occurs in various situations, from contamination threats (e.g., mold) to moral violations (e.g., murder). Like fear, disgust is negative, arousing, occurs during and following trauma, and predicts posttraumatic stress (PTS) symptoms (Matson et al., 2023). We know people attend to, and remember, disgust stimuli more than fear (Moeck et al., 2021). But does remembering disgust *more* mean remembering disgust *better* than fear? To answer this question, we need to establish that people are less likely to *falsely* remember disgust (than fear) stimuli. Existing research (e.g., Chapman et al., 2013) finds consistently low—and similar—false-memory rates for disgust and fear, likely because ‘new’ image lures were unrelated to encoded ‘old’ images, among other limitations. Therefore, we compare disgust and fear false-memory rates when test images are *related* (and unrelated) to encoded images.

People remember emotional—highly arousing and negative (or positive)—stimuli more than neutral stimuli (*emotionally enhanced memory*; Cahill & McGaugh, 1995). From an evolutionary standpoint, remembering stimuli that elicit emotion can be important for human survival (Darwin, 1872/1965). For example, remembering *disgust*-eliciting stimuli protects us from threats of contamination (e.g., contact with bodily fluids), making disgust a central emotion in the ‘behavioral immune system’ (Schaller & Park, 2011). By contrast, remembering *fear*-eliciting stimuli protects us from imminently dangerous situations (e.g., escaping a predator; Ekman, 1992). However, disgust and fear stimuli are not remembered equally (Moeck et al., 2021).

Emotional conditioning theories (e.g., Olatunji et al., 2007) suggest people learn disgust and fear responses differently, which may explain memory differences between the two emotions. Disgust and fear reactions are formed through classical conditioning (i.e., pairing an unconditioned stimulus with a conditioned stimulus, which produces the conditioned emotional response) and operant conditioning (i.e., avoiding stimuli that elicit the emotional response reinforces future avoidance) processes (Badour & Feldner, 2018). One classical conditioning process is *expectancy learning*, where, in the context of disgust and fear conditioning, people acquire these emotional responses due to learning that the conditioned stimulus predicts a threatening outcome (Reynolds & Askew, 2021). For example, a person who consumes spoiled meat and develops food poisoning may then feel *disgust* towards, and avoid, that meat to avoid future contamination. Turning to fear, a person who is attacked by a dog may then feel *fear* towards, and avoid, dogs to prevent being attacked in the future. These conditioned emotional responses can be reduced—or even extinguished—through *exposure* (i.e., ceasing avoidance behaviors); if a person consumes nonspoiled meat, or if a person approaches a well-trained dog, they then learn that the expected threat does not usually occur.

However, disgust is not always sufficiently reduced via exposure (Mitchell et al., 2024) because disgust reactions can also form via *evaluative conditioning* (Olatunji et al., 2007). In evaluative conditioning, an affective value (e.g., disgust) is placed onto the conditioned stimulus, even if there is no expectation that the unconditioned stimulus will occur again (De Houwer et al., 2001). In the context of traumatic experiences, evaluative conditioning may involve transferring disgusting aspects of the experience onto the self or others (Badour & Feldner, 2018). For example, a police officer investigating child exploitation may view themselves as disgusting for witnessing such content. Once a stimulus or person is labelled as disgusting, its disease and/or violation properties are difficult to change or eliminate (Harned et al., 2015), suggesting emotions elicited via evaluative conditioning (i.e., *disgust*) may be particularly memorable.

In line with this idea, people freely recall more disgust-eliciting than fear-eliciting images (termed *disgust memory enhancement*), even when these images are matched on memory-enhancing variables like arousal (i.e., alertness), valence (i.e., emotional positivity or negativity), distinctiveness (i.e., eye-catching/unusualness) and/or interrelatedness (i.e., the conceptual similarity of images within a stimulus set; Chapman, 2018; Chapman et al., 2013; Moeck et al., 2021; Schienle et al., 2021). Notably, remembering *more* disgust than fear is not the same as remembering disgust *better*—or more accurately—than fear. For example, someone may recall seeing a bloody leg, but may not accurately identify which (of two) bloody legs they previously saw. Alternatively, someone may recall seeing disgust images depicting different content to what they originally saw. In both cases, remembering *more* does not translate to *better* memory for disgust. Therefore, we wondered whether disgust memory enhancement extends to *accurate memory.*

The prevailing explanation for disgust memory enhancement is that people better encode disgust stimuli because they pay more attention to disgust than fear (Carretié et al., 2011). Across eye-tracking (Fink-Lamotte et al., 2021) and behavioral (Chapman et al., 2013) measures, disgust captures and holds attention more than fear. However, while some studies find that disgust’s attentional salience fully (Chapman, 2018) or partially (Chapman et al., 2013) accounts for disgust memory enhancement, other studies find no evidence for this idea (Matson et al., 2024; Moeck et al., 2021). Given this mixed evidence, perhaps *retrieval* mechanisms contribute to disgust memory enhancement. Specifically, people may be susceptible to disgust-specific *memory amplification* (Oulton et al., 2016; Southwick et al., 1997), potentially retrieving more true but also more *imagined* (false) details of disgust than fear stimuli. If so, disgust memory enhancement should result in more *false memories*. Because both encoding (attention) and retrieval (memory amplification) mechanisms may contribute to disgust memory enhancement, we consider competing hypotheses for disgust versus fear false-memory rates.

Perhaps people have *fewer* false memories for disgust than fear. Carretié et al.’s (2011) cost-and-benefit hypothesis considers the evolutionary functions of disgust and fear, and how these functions may differentially influence how closely a person attends to disgust- or fear-inducing stimuli. This hypothesis posits that people maintain attention on disgust stimuli for longer than fear to ‘explore’ costs and benefits. Exploring fear stimuli is costly because these stimuli require imminent/urgent action. Exploring disgust stimuli (e.g., contaminated food) is less costly because these stimuli are not imminently dangerous. Further, disgust stimuli are typically ambiguous, meaning exploring such stimuli has benefits (e.g., revealing the food is consumable). Therefore, people should have better memory sensitivity for disgust (i.e., can better distinguish disgust images they have/have not encoded), resulting in *fewer* false memories for disgust than fear.

However, there are two reasons why people may have *more* false memories for disgust than fear. First, although disgust stimuli capture people’s attention, they may superficially explore disgust stimuli (i.e., attend but avoid encoding specific information; termed *attentional rubbernecking*), resulting in a weak memory trace (Fink-Lamotte et al., 2021). Second, the extended exploration and/or ambiguous nature of disgust may lead people to generate additional, imagined details about these stimuli (memory amplification; e.g., seeing an image of a dirty toilet and remembering a different dirty toilet). This process could increase feelings of familiarity toward related disgust stimuli presented at test, reducing memory sensitivity and making participants more likely to judge such stimuli as ‘old’ (i.e., experience *source monitoring errors*; Lindsay & Johnson, 2000) compared with related fear stimuli.

Alternatively, these predictions might counteract, resulting in *similar* false-memory rates for disgust and fear. Research supports this possibility: people correctly recognize (i.e., respond ‘old’ to test images seen at encoding) disgust images more than fear images but falsely remember (i.e., respond ‘old’ to test images *not* seen at encoding) a similar, low proportion of disgust and fear images (Chapman et al., 2013; Croucher et al., 2011; Marchewka et al., 2016; Schienle et al., 2021). But there are limitations to these studies.

In two studies, researchers used unrelated test image “lures” from broad emotion *categories* to capture false memories (Chapman et al., 2013; Marchewka et al., 2016). For example, showing a slug at encoding then a dirty toilet at test. Such *unrelated* images likely make the recognition test easy (Bowman & Dennis, 2015); participants can confidently reject new/unseen images, resulting in low false-memory rates. Giving participants the opportunity to falsely remember related—but not previously seen—images at test (e.g., showing different slugs at encoding and test; Schienle et al., 2021) should increase false-memory rates, allowing us to reliably determine whether disgust memory enhancement extends to *accurate* memory.

In two other studies, researchers included related test lures: each ‘old’ image was paired with a ‘lure’ image depicting similar content (Croucher et al., 2011; Schienle et al., 2021). These studies also resulted in similarly low disgust and fear false-memory rates. But in neither study were ‘old’ and ‘lure’ image pairs normed on similarity/relatedness—that is, how closely the images resembled one another. Thus, whilst conceptually similar, it is unclear whether these old/lure image pairs *looked* similar enough to make the recognition test difficult. These studies have other limitations. A G*Power sensitivity analysis indicated Croucher et al.’s (2011) sample size (*N* = 32) was insufficient to detect small effect sizes (η_p_^2^ < .05) for a repeated-measures analysis of variance (ANOVA), while Schienle et al. (2021) did not match their disgust and fear image sets on memory-enhancing variables (arousal, valence, distinctiveness). We address these limitations by matching old/lure image pairs on similarity, matching disgust and fear image sets on memory enhancing variables,^1^ and recruiting a sufficient sample.

## The current study

We tested our competing hypotheses by examining false-memory rates for disgust versus fear images using related *and* unrelated lures (to examine whether related lures increase false-memory rates). Participants encoded disgust, fear, and neutral images whilst completing a task measuring attentional capture (Chapman et al., 2013). After 24–48 hours, participants completed a recognition test including ‘old’ (previously seen) and ‘new’ (not previously seen, related and unrelated) images. Along with our competing hypotheses, we expected participants would attend to disgust more than fear and neutral images at encoding (Matson et al., 2024), and correctly recognize more disgust than fear and neutral images at test (Chapman et al., 2013; Schienle et al., 2021).

We also examined response bias (tendency to say ‘old’ or ‘new’). Due to mixed evidence on response bias differences between disgust and fear (Boğa et al., 2021), we aimed to clarify whether participants have a more—or similarly—liberal response bias for disgust versus fear with no directional hypothesis. We had three exploratory interests: confidence in old/new judgements for lures, remember/know judgements to lures identified as ‘old’, and correlations between memory for disgust, trait disgust, and PTS symptoms. Examining whether people are prone to falsely remember disgust (and fear) is clinically important: People feel both emotions during/following trauma (Badour & Feldner, 2018), and falsely remembering previously unseen images (a form of memory amplification) correlates with worsening PTS symptoms in relation to those images (Oulton et al., 2016).

## Method

We preregistered this study (https://osf.io/vbs9w); data (https://osf.io/7v6ax) and supplementary material (https://osf.io/bt3ak) are publicly available. We report how we determined sample size and all data exclusions, manipulations, and measures in the study (Simmons et al., 2012). The Flinders University Social and Behavioral Research Ethics Committee approved this research.

### Participants

Brysbaert (2019) suggests psychological research findings start to have practical and/or theoretical relevance at a medium effect size (*d* = .40). Thus, we based our target *n* = 110 on Brysbaert’s recommendation for a 2 × 2 within-subjects design (our main analysis of interest) and predicting an interaction, using *d* = .40 with 80% power. We took the following preregistered steps to ensure quality data. To prevent bots/server farmers from completing the surveys, participants had to pass prescreening questions: a reCAPTCHA, an arithmetic question (i.e., 18 + 7 =) presented as an image, and score at least 8/10 on an English proficiency test (Moeck et al., 2022) to start the surveys. We embedded two attention checks (e.g., ‘If you are reading this, please select response 7’) and two open-ended questions (e.g., ‘Please describe the image that captured your attention the most’) within the surveys. We recruited 131 participants living in the USA from Amazon Mechanical Turk (MTurk) via CloudResearch (Litman et al., 2017) but excluded 20: One participant completed the encoding task twice, nine participants did not follow instructions for the encoding task, nine participants did not complete the recognition test, and one participant completed the recognition test too late (>72 hours after the encoding task).

Our final sample comprised 111 participants ranging in age from 24 to 78 years (*M* = 44.6, *SD* = 12.4); most were men (64.9%, women = 33.3%, nonbinary = 0.9%, prefer not to say = 0.9%). Most participants were White/Caucasian (75.7%); other participants were Black/African American (9.9%), Latino/Hispanic (4.5%), East Asian (4.5%), Mixed Race (1.8%; as noted in the ‘other’ option box), South Asian (0.9%), South-East Asian (0.9%), Mixed/Asian (0.9%), and one participant preferred not to disclose their ethnicity.

### Materials

#### Image set development

We conducted several pilot studies to match our disgust and fear image sets on various memory-enhancing variables and ensure they evoked their target emotion. We first obtained ratings of disgust, fear, arousal, pleasantness, unpleasantness, and distinctiveness for potential disgust, fear, and neutral images taken from several sources: the Nencki Affective Picture System (NAPS; Marchewka et al., 2014), a study by Grootswagers et al. (2020), a study by Chapman (2018), the Disgust Related Images database (DIRTI; Haberkamp et al., 2017), the Socio-Moral Image Database (SMID; Crone et al., 2018), the EmoMadrid database (Carretié et al., 2019), the Open Affective Standardized Image Set (OASIS; Kurdi et al., 2016), and the Crime and Threat Image Set (CaTIS; Noon et al., 2019). In total, 611 MTurk participants (53.0% women, 45.0% men, 1.1% nonbinary, 0.7% ‘prefer not to say’, and 0.2% ‘other’) ranging in age from 20 to 75 years (*M*_age_ = 41.5 years, *SD*_age_ = 12.0) rated between 21 and 53 images each (from a pool of 362 images), until we had at least 50 ratings on each dimension per image. We excluded 147 images that were rated too high on the alternate emotion (e.g., disgust images rated >4 on fear) and/or too low on the target emotion (e.g., disgust images rated <4 on disgust), leaving 215 images.

From these eligible images, we created 333 pairs consisting of either two disgust or two fear images that had related content (e.g., there were 14 potential pairings of sharks). These pairs represented 20 distinguishable disgust themes (e.g., garbage, surgery, vomit) and 19 distinguishable fear themes (e.g., motor vehicle accident, snake, gun pointed at the screen). Next, to ensure the images in each pair were visually related to one another (Heathcote et al., 2009), a total of 250 MTurk participants (54.4% women, 44.4% men, 1.2% nonbinary, and 0.4% ‘prefer not to say’) ranging in age from 21 to 76 years (*M*_age_ = 42.9 years, *SD*_age_ = 11.5) rated image pairs on similarity (1 = *not similar at all*, 5 = *very similar*). Across several phases of piloting,^2^ participants rated a selection of potential pairs (from the 333 eligible image pairs) presented vertically/top to bottom.

#### Images

Based on our pilot testing, we selected 36 disgust images (i.e., 12 pairs and 12 single unrelated images; rated high on disgust/low on fear) and 36 fear images (i.e., 12 pairs and 12 single unrelated images; rated high on fear/low on disgust). The unrelated images depicted different themes to the image pairs (e.g., seeing a dirty toilet at test, but not encoding). Of the disgust and fear image pairs, we only selected pairs rated high on similarity (>3) and ensured the image sets were comparable overall in similarity ratings (disgust image pairs: *M* = 4.1, *SD* = 0.9; fear image pairs: *M* = 4.1, *SD* = 0.8). Disgust images included injuries/deformity, death, mold, garbage, body products, dirty objects, and nonthreatening animals. Fear images included threatening animals, weapons, scary faces, disasters in progress, and human attacks. See https://osf.io/3d67w for image codes and Supplementary Table 1 for a full list of themes depicted in our image sets.

**Fig. 1:**
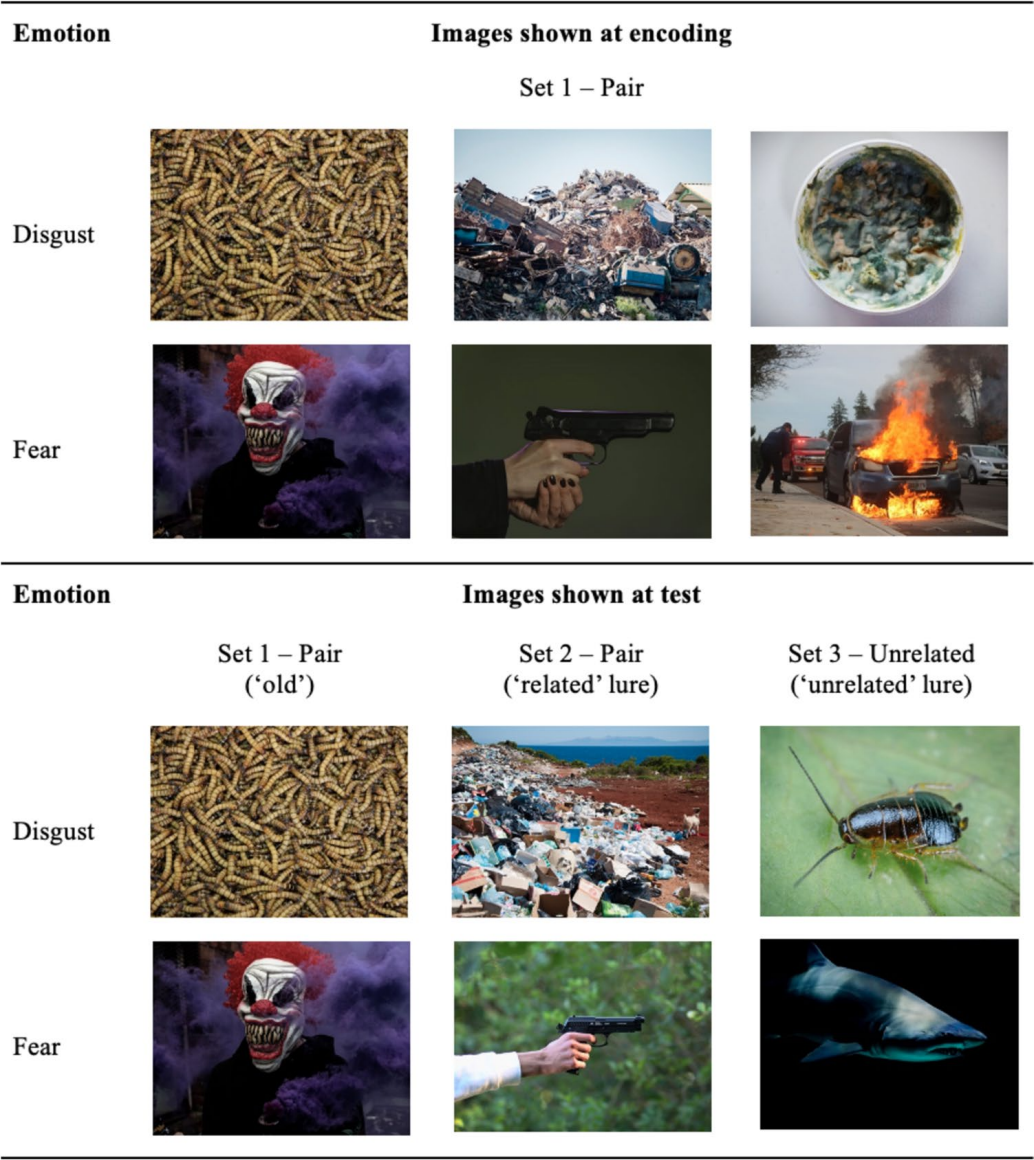
Examples of disgust and fear image themes shown to participants during the encoding and test phases. *Note.* Here, images in ‘Set 1–Pair’ were shown both at encoding and at test, test images in ‘Set 2–Pair’ depict related pairs of images shown at encoding (from Set 1–Pair), and test images in ‘Set 3–Unrelated’ depicts content unrelated to what was shown at encoding. These public domain images are examples only and were not part of our image sets. Disgust and fear image themes depicted in our image sets are presented in Supplementary Table 1. (Color figure online)

To eliminate order effects, we created sets of images and counterbalanced between participants so that some images were seen at encoding, and others at test. Specifically, we categorized disgust and fear images into three sets per emotion category: Set 1–Pair (containing 12 images that had a corresponding pair), Set 2–Pair (containing the 12 corresponding images to Set 1–Pair) and Set 3–Unrelated (containing 12 unrelated images). During encoding, participants viewed the disgust and fear images in either Set 1–Pair *or* Set 2–Pair. During test, participants viewed six disgust and six fear images from each of the three image sets. For example, among the three disgust sets participants viewed six ‘old’ images (half of the images that they viewed at encoding), six ‘related lure’ images (half of the images in the alternate pair image set), and six ‘unrelated lure’ images (half of the images in Set 3–Unrelated). Figure 1 shows an example of images shown to participants at encoding versus test.

All six image sets (three disgust, three fear) were matched as closely as possible on arousal, pleasantness, unpleasantness, and distinctiveness. Table 1 displays descriptive statistics for the disgust and fear image ratings overall.^3^ To test whether the disgust and fear image sets were matched on arousal, pleasantness, unpleasantness, and distinctiveness and differed on disgust and fear, we used the item analysis approach (like Chapman et al., 2013) where images are treated as ‘subjects’ (and each mean ratings score was averaged across all participants who rated that image). Participants rated the disgust and fear images as similarly distinctive, *t*(70) = −1.52, *p* = .13, *d* = −0.36. Fear images were significantly more arousing than disgust images (*t*(70) = −3.32, *p* = .001, *d* = −0.78), though this difference was small (*M*_diff_ = 0.5). Disgust images were significantly more unpleasant (*t*(70) = 3.49, *p* < .001, *d* = 0.82), and less pleasant (*t*(70) = −3.40, *p* = .001, *d* = −0.80), than fear images, though, again, these differences were small (*M*_diff_ = 0.4, *M*_diff_ = 0.2, respectively). Disgust and fear images differed on disgust (*t*(70) = 15.69, *p* < .001, *d* = 3.70) and fear (*t*(70) = −12.58, *p* < .001, *d* = −2.97) ratings, in the expected direction. The arousal and valence (i.e., pleasantness and unpleasantness) differences shown in our disgust and fear image sets are consistent with previous research (e.g., Chapman et al., 2013; Matson et al., 2024) and thus likely reflect real-world differences between disgust and fear characteristics that are difficult to eliminate.

**Table 1:** Ratings—*M* (*SD*)—for the disgust and fear images

Rating type (*scale*)	Disgust	Fear
Arousal	3.5 (0.7)	4.0 (0.4)
Pleasantness	1.3 (0.2)	1.5 (0.3)
Unpleasantness	5.4 (0.6)	5.0 (0.4)
Distinctiveness	4.5 (0.8)	4.7 (0.5)
Disgust	5.0 (0.6)	2.7 (0.6)
Fear	2.9 (0.8)	4.6 (0.3)

*Note.* Likert scales are as follows: Arousal (1 = *not at all arousing*, 7 = *highly arousing*); Pleasantness (1 = *not at all pleasant*, 7 = *extremely pleasant*); Unpleasantness (1 = *not at all unpleasant*, 7 = *extremely unpleasant*); Distinctiveness (1 = *not at all distinctive*, 7 = *extremely distinctive*); Disgust (1 = *not at all disgusting*, 7 = *extremely disgusting*); Fear (1 = *not at all frightening*, 7 = *extremely frightening*). Descriptive statistics presented in Table 1 reflect all disgust images and all fear images collapsed into one set per emotion category. Descriptive statistics for disgust and images grouped per set (i.e., two image pair sets and one unrelated images set, per emotion category) are presented in Supplementary Table 2

Regarding the neutral images, we selected 24 images displaying conceptually different (i.e., unrelated) content, rated low on all dimensions, except intermediate on pleasantness, *M* = 4.5, *SD* = 0.8. Neutral images^4^ included common objects and everyday scenes.

#### Encoding task: Line discrimination

We used a line discrimination task (LDT) to measure attention toward each image during encoding. As in previous studies (e.g., Moeck et al., 2021), all 36 images appeared in a random order, for 2 s each, accompanied by a horizontal white line 0.5 cm above or below the image. We instructed participants to indicate the line’s position as quickly as possible via key press, with slower responses suggesting greater attention captured by each image. The LDT indirectly measures how much a person’s attention is captured by an image. The LDT follows the same logic as other exogenous measures of attention (e.g., the emotional Stroop task), which are widely accepted as a valid index of attentional capture (Carretié, 2014). In the current study, slower reaction times when the line co-occurs with disgust relative to fear images suggests that disgust images are more attentionally salient (i.e., participants take longer to respond to the line due to staring at the disgust images for longer instead; Chapman et al., 2013). The image remained visible for the remaining time after the participant’s response (e.g., 1,500 ms for a 500-ms response).

#### Trait disgust

We measured trait disgust—disgust sensitivity, disgust propensity, and disgust avoidance behaviors—via two scales.

The *Disgust Propensity and Sensitivity Scale–Revised* (DPSS-R; Fergus & Valentiner, 2009) measures disgust propensity (six items; e.g., ‘I avoid disgusting things’) and disgust sensitivity (six items; e.g., ‘When I feel disgusted, I worry that I might pass out’; *1 = never, 5 = always*). The DPSS-R had good internal consistency (current study: propensity α = .83, sensitivity α = .85).

The 17-item *Disgust Avoidance Questionnaire* (DAQ; von Spreckelsen et al., 2022) measures people’s tendency to avoid experiencing disgust and comprises three subscales: Disgust Prevention (e.g., ‘I try hard to avoid situations that might bring up feelings of repulsion in me’), Cognitive Disgust Avoidance (e.g., ‘I try hard to avoid thinking about a repulsive past situation’), and Behavioral Disgust Avoidance (e.g., ‘I am quick to leave any situation that makes me feel disgusted’; 1 =* strongly disagree, *7 = *strongly agree*). The DAQ had high internal consistency (current study: α = .97).

#### Posttraumatic Stress Disorder Checklist (PCL-5; Blevins et al., 2015)

Participants answered the PCL-5 in relation to their most traumatic/stressful life event. We did not measure whether participants’ traumatic/stressful event met Criterion A (as defined in the *Diagnostic and Statistical Manual for Mental Disorder*’s PTSD diagnostic criteria; American Psychiatric Association, 2022) because traumatic stress reactions also exist in response to non-Criterion A events (Bridgland et al., 2021; Georgescu et al., 2024). The PCL-5 comprises 20 items, including four subscales measuring key symptom clusters (Re-experiencing, Avoidance, Negative Alterations in Cognition and Mood, Alterations in Arousal and Reactivity). Items (e.g., ‘Repeated, disturbing, and unwanted memories of the stressful experience’) are rated from 0 = *not at all* to 4 = *extremely*. The PCL-5 had high internal consistency (current study: α = .96).

### Procedure

#### Encoding phase

After passing prescreening (reCAPTCHA, arithmetic question, English proficiency test), participants provided demographic information (gender, age, ethnicity). They then completed the LDT, where they encoded 12 disgust, 12 fear (Set 1–Pair *or* Set 2–Pair; set allocation was randomized across participants), and 12 neutral images whilst indicating the location of a line (presented either above or below each image). Next, participants reported how closely they attended to the images (1 = *not at all closely*, 7 = *extremely closely*), whether they closed their eyes or looked away from the images (*yes*/*no*), and if *yes*, for approximately how many images.^5^

#### Test phase

Following a 24–48-hour delay^6^ (*M* = 30.2 hours, *SD* = 8.6 hours), participants completed a recognition test, where they viewed previously encoded (‘old’) and never seen (‘new’; lures) disgust, fear, and neutral images; the new disgust and fear images comprised both related (i.e., the image from the image pair that was not viewed during encoding) and unrelated images. Overall, at test, participants viewed 48 images in a randomized order comprising: six old images per emotion category, six related image lures (only for disgust and fear), and six unrelated image lures per emotion category. Participants indicated whether each image was ‘old’ (i.e., previously seen) or ‘new’ (i.e., not previously seen). Immediately after each old/new decision, participants rated their confidence in their answer (1 =* not at all*, 5 = *very*). If participants indicated an image was ‘old’, they made a remember/know judgement. Participants were told that ‘remember’ means they recognized the image as one they had seen during the encoding phase and ‘know’ means the image seemed familiar but they did not explicitly recall viewing the image during the encoding phase (full instructions for the recognition test are reported in supplementary material). After the recognition test, participants completed the PCL-5, and then the two disgust scales (DPSS-R and DAQ, in a randomized order), and were debriefed. Participants received US $3.00; the study took approximately 30-min across both sessions to complete (Time 1: ~10 min; Time 2: ~20 min).

## Results

### Descriptive statistics

Table 2 displays descriptive statistics for variables used in the main analyses.

**Table 2:** Descriptive statistics for main variables by image emotion category (disgust, fear, neutral)

Variables	Disgust *N*	Disgust *M* (*SD*)	Fear *N*	Fear *M* (*SD*)	Neutral *N*	Neutral *M* (*SD*)
**Related lures**						
False-memory rates (proportion)	111	.32 (.26)	111	.30 (.26)	–	–
Memory sensitivity (*d*’)	109	1.3 (0.9)	107	1.1 (0.8)	–	–
Response bias (*c*)	109	−0.2 (0.9)	107	0.2 (1.1)	–	–
Confidence ratings (percentage)	111	72.8 (16.3)	111	69.0 (18.4)	–	–
Remember (proportion)	87	.47 (.41)	83	.42 (.43)	–	–
Know (proportion)	87	.53 (.41)	83	.58 (.43)	–	–
**Unrelated lures**						
False-memory rates (proportion)	111	.13 (.21)	111	.22 (.23)	111	.10 (.20)
Memory sensitivity (*d*’)	110	1.8 (0.7)	109	1.3 (0.9)	–	–
Response bias (*c*)	110	0.3 (0.9)	109	0.4 (1.0)	–	–
Confidence ratings (percentage)	111	75.8 (19.6)	111	71.1 (19.0)	–	–
Remember (proportion)	46	.41 (.46)	68	.38 (.43)	–	–
Know (proportion)	46	.59 (.46)	68	.62 (.43)	–	–
**Correction recognition rates (proportion)**	111	.76 (.21)	111	.65 (.26)	111	.36 (.27)
**LDT response times (ms)**	111	646.9 (170.3)	111	629.6 (150.6)	111	609.3 (137.3)

*Note. N* values vary due to missing data for *d′* and *c* and because participants only provided remember/know ratings for images they identified as ‘old’ (i.e., only 87 participants misjudged at least one related disgust lure as ‘old’ and thus, the remaining 24 participants correctly rejected all related disgust lures as ‘new’

### Preliminary analyses

Prior to examining our main research question relating to false memories, we first examined differences in attention and correct recognition among disgust, fear, and neutral images (see Table 2 for descriptive statistics).^7^ First, we tested whether the disgust images captured participants’ attention (i.e., they responded slower to the line during the LDT, in ms) more than the fear and neutral images. A one-way repeated-measures ANOVA on LDT response times^8^ revealed a significant effect of emotion category, *F*(2,220) = 17.97, *p* < .001, η_p_^2^ = .14. Participants showed slower LDT responses when the line co-occurred with disgust compared with fear (*M*_diff_ = 17.4; 95% CI [4.4, 30.3], *p* = .009, *d* = 0.11) and neutral (*M*_diff_ = 37.7; [24.1, 51.2], *p* < .001, *d* = 0.24) images. Further, participants showed slower LDT responses when the line co-occurred with fear compared with neutral images (*M*_diff_ = 20.3; [9.6, 31.1], *p* < .001, *d* = 0.14). Thus, replicating previous studies (Chapman, 2018; Chapman et al., 2013; Matson et al., 2024; Moeck et al., 2021; van Hooff et al., 2013), the disgust images captured participants’ attention longer than the fear and neutral images.

**Fig. 2:**
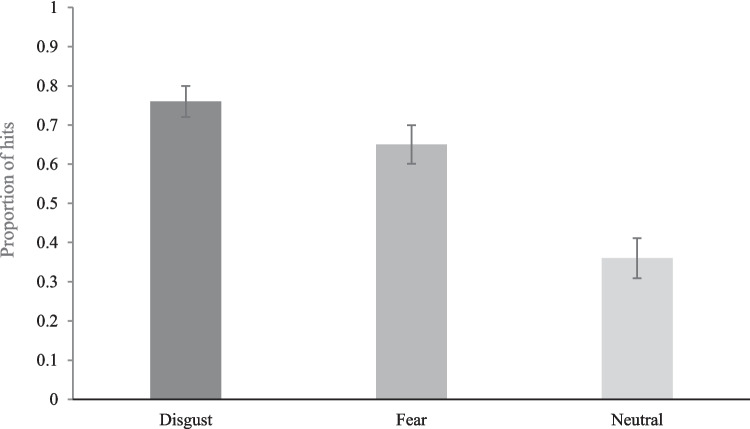
Mean proportion of correctly recognized disgust, fear and neutral images (i.e., hits), with 95% confidence intervals (Masson & Loftus, 2003)

Next, we tested whether participants correctly recognized (i.e., hits) a higher proportion of disgust than fear—and fear than neutral—previously seen (‘old’) images. As Fig. 2 shows, a one-way repeated-measures ANOVA revealed a significant effect of emotion category on correction recognition rates, *F*(2,220) = 109.76, *p* < .001, η_p_ = .50. Simple contrasts revealed participants correctly recognized more disgust than fear (*M*_diff_ = .10; 95% CI [.05, 1.0], *p* < .001, *d* = 0.47) and neutral (*M*_diff_ = .39; [.34, .45], *p* < .001, *d* = 1.65) images. Further, participants correctly recognized more fear than neutral (*M*_diff_ = .29; [.24, .34], *p* < .001, *d* = 1.09) images. Thus, consistent with previous research (e.g., Schienle et al., 2021), participants correctly recognized more disgust than fear—and more fear than neutral—images, which suggests disgust memory enhancement extends to more *accurate* memory.

### Main analyses

**Fig. 3:**
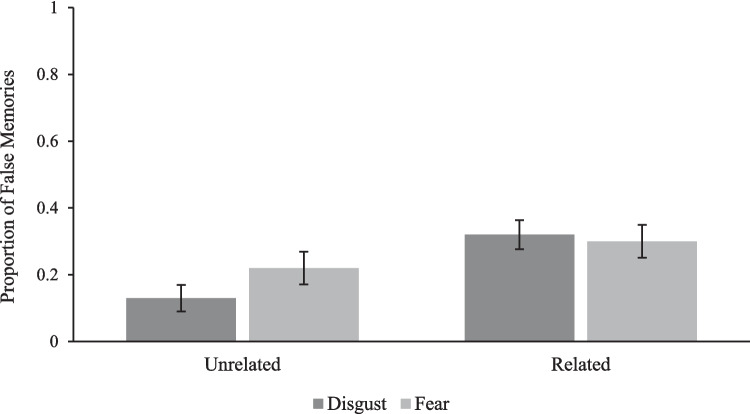
Mean proportion of disgust and fear false memories by lure type (related, unrelated), with 95% confidence intervals (Masson & Loftus, 2003)

Recall our primary aim was to determine whether participants had more, fewer, or a similar number of false memories for disgust relative to fear images. Hereon, we omit neutral images from analyses and test the effects of emotion category (disgust, fear) and image lure type (related, unrelated) on memory recognition. As Fig. 3 shows, a two-way repeated-measures ANOVA revealed a statistically significant interaction between emotion (i.e., disgust, fear) and image lure type (i.e., related, unrelated) on false-memory rates, *F*(1,110) = 11.40, *p* = .001, η_p_^2^ = .094. Simple contrasts revealed a statistically significant difference between disgust and fear false memories for unrelated lures (*M*_diff_ = .09; 95% CI [.05, .12], *p* < .001, *d* = 0.41) but not for related lures (*M*_diff_ = .02; [−.03, .07], *p* = .40, *d* = 0.08). Consistent with the cost-and-benefit hypothesis (Carretié et al., 2011), participants had *fewer* false memories of disgust compared with fear images overall; a significant main effect of emotion category, *F*(1,110) = 5.38, *p* = .02, η_p_^2^ = .05. Furthermore, participants had more false memories of related compared with unrelated lures; a significant main effect of lure type, *F*(1,110) = 77.61, *p* < .001, η_p_^2^ = .41. Thus, these findings suggest people are less susceptible to falsely remembering disgust compared with fear stimuli, but when there is a high likelihood of experiencing source monitoring errors (i.e., when stimuli are closely related), people are similarly susceptible to falsely remembering disgust and fear stimuli. We further test this idea by examining memory sensitivity for disgust relative to fear images (and for both lure types).

We used a signal detection approach (i.e., correctly classifying an old image as ‘old’ is a hit, incorrectly classifying a new image as ‘old’ is a false alarm; Stanislaw & Todorov, 1999) to calculate memory sensitivity (*d′*) and response bias (*c*). Descriptive statistics of *d′* and *c* for images in each emotion/lure type category appear in Table 2.

We first turn to memory sensitivity. A *d′* value of zero indicates an inability to accurately distinguish between previously seen and unseen images, and larger values indicate an increased ability to accurately distinguish between previously seen and unseen images and thus more genuine recognition. A two-way repeated-measures ANOVA revealed a statistically significant interaction between emotion (disgust, fear) and image lure type (related, unrelated) on memory sensitivity, *F*(1,103) = 15.70, *p* < .001, η_p_^2^ = .13. Simple contrasts showed a statistically significant difference between memory sensitivity for disgust and fear images, when comparing previously seen (old) images with new related lure images (*M*_diff_ = 0.2; 95% CI [0.04, 0.4], *p* = .02, *d* = 0.23) and new unrelated lure images (*M*_diff_ = 0.6; [0.4, 0.8], *p* < .001, *d* = 0.62). Also consistent with the cost-and-benefit hypothesis (Carretié et al., 2011), participants had *better* memory sensitivity for disgust compared with fear images; a significant main effect of emotion category, *F*(1,103) = 22.56, *p* < .001, η_p_^2^ = .18. Furthermore, participants had *poorer* memory sensitivity for related compared with unrelated lures; a significant main effect of lure type, *F*(1,103) = 69.12, *p* < .001, η_p_^2^ = .40. Our findings thus suggest participants’ memory of disgust (relative to fear) images, and unrelated (relative to related) lures, was more accurate (i.e., they were better able to distinguish between images they had vs. had not seen before).

Next, we turn to response bias for disgust relative to fear images (both lure types), where *c* < 0 indicates a bias toward identifying test items as old (i.e., liberal response bias) and *c* > 0 indicates a bias toward identifying test items as new (i.e., conservative response bias). A two-way repeated-measures ANOVA revealed a statistically significant interaction between emotion (disgust, fear) and lure type (related, unrelated) on response bias, *F*(1,103) = 15.70, *p* < .001, η_p_^2^ = .13. Simple contrasts showed a statistically significant difference between response bias for disgust and fear images, when comparing previously seen (old) images with new related lure images (*M*_diff_ = 0.4; 95% CI [0.2, 0.6], *p* < .001, *d* = 0.40), but not when comparing old images with new unrelated lure images (*M*_diff_ = 0.04; [−0.1, 0.2], *p* = .68, *d* = 0.08). Participants had a more liberal response bias for disgust compared with fear images; a significant main effect of emotion category, *F*(1,103) = 5.98, *p* = .02, η_p_^2^ = .06. Furthermore, participants had a more conservative response bias for unrelated compared with related lures; a significant main effect of lure type, *F*(1,103) = 69.12, *p* < .001, η_p_^2^ = .01. Therefore, people make more liberal judgements for disgust relative to fear stimuli when there is a high likelihood of experiencing source monitoring errors (i.e., when stimuli are closely related). When source monitoring errors are less likely to occur (i.e., when stimuli are unrelated), people make similarly conservative judgements for disgust and fear stimuli.

### Exploratory analyses

We had three exploratory interests. First, we wondered whether participants’ confidence in their old/new judgements differed for disgust and fear (as well as related and unrelated) image lures. A two-way repeated-measures ANOVA revealed a nonsignificant interaction between emotion (disgust, fear) and image lure type (related, unrelated) on confidence ratings, *F*(1,110) = 0.33, *p* = .57, η_p_^2^ = .003. However, participants were more confident in their judgements for disgust than fear images (a significant main effect of emotion, *F*(1,110) = 24.96, *p* < .001, η_p_^2^ = .19) and in their judgements for unrelated than related image lures (a significant main effect of lure type, *F*(1,110) = 8.91, *p* = .003, η_p_^2^ = .08). These results suggest participants were relatively aware of their memory ability; their confidence in their judgements translated to actual performance (i.e., participants had better memory sensitivity for disgust relative to fear images, and for unrelated relative to related image lures). When recognition was more difficult—as in, the likelihood of experiencing source monitoring errors increased—people were less confident in their responses.

Second, we wondered whether participants made different ‘remember’ (i.e., vividly remember viewing the image) versus ‘know’ (i.e., the image is familiar, though they do not explicitly remember viewing the image) judgements to the disgust and fear image lures they identified as ‘old’ (i.e., false memories). Higher ‘remember’ judgements for false memories of a specific emotion category and/or lure type would indicate a weaker memory trace and increase in source monitoring errors for that emotion category/lure type (Holmes et al., 1998). A two-way repeated-measures ANOVA revealed a nonsignificant interaction between emotion (disgust, fear) and image lure type (related, unrelated) on remember responses, *F*(1,37) = 0.01, *p* = .92, η_p_^2^ = .0003. There were also no significant main effects, *F*(1,37) = 0.17, *p* = .68, η_p_^2^ = .01, for emotion; *F*(1,37) = 0.54, *p* = .47, η_p_^2^ = .01, for lure type. Because only a subset of participants (*n* = 38) experienced false memories of both related and unrelated lures—and thus only this subset gave remember/know ratings for images identified as ‘old’ in the above analyses—these results are underpowered and should be interpreted with caution. Nevertheless, these findings suggest participants similarly subjectively remembered the disgust and fear lure images.

Finally, we wondered whether memory for disgust (i.e., correct recognition, false memories, memory sensitivity, and response bias) correlated with trait disgust and PTS symptoms. These data and descriptive statistics for trait disgust and PTS symptoms are reported in Supplementary Tables 5 and 6. Overall, memory for disgust did not significantly correlate with trait disgust (i.e., scores on the DPSS-R or DAQ) or PTS symptoms (i.e., scores on the PCL-5). These findings should be interpreted with caution since our sample size is likely too small to detect stable correlations (Schönbrodt & Perugini, 2013, 2018).

## Discussion

We aimed to determine whether people experience fewer, similar, or more false memories for disgust versus fear images. We addressed past research limitations (e.g., Chapman et al., 2013) by using related *and* unrelated image lures and well-matched image sets. When image lures depicted content *unrelated* to ‘old’ images, participants falsely remembered fewer disgust than fear images. However, when image lures depicted content *related* to ‘old’ images, participants falsely remembered disgust and fear at a similar rate, but had a more liberal response bias for disgust than fear. Regardless of lure type, participants correctly recognized more disgust than fear images, and had better memory sensitivity for disgust than fear images. Together, these findings suggest disgust memory enhancement results in fewer false memories for disgust. Put otherwise, disgust memory enhancement extends to remembering disgust *more accurately* than fear.

In line with the cost-and-benefit hypothesis (which considers disgust and fear’s evolutionary functions; Carretié et al., 2011), the disgust images captured participants’ attention more than the fear images (as in Matson et al., 2024), and participants falsely remembered fewer disgust than fear images. Perhaps because of disgust’s ambiguous nature, participants explored—and encoded—disgust images longer than fear images, resulting in better memory for disgust. Consistently, participants had better memory sensitivity for disgust than fear images, even among related lures (where participants falsely remembered similar rates of disgust and fear related lures). Here, accurate memory for disgust was driven by participants’ ability to correctly recognize more ‘old’ disgust than fear images (rather than falsely remember fewer disgust than fear images). These findings disconfirm the proposition that people only superficially explore disgust stimuli, leading to a weak memory trace (Fink-Lamotte et al., 2021).

Our finding that participants falsely remembered fewer—and for related lures, similar—disgust than fear images did not support our hypothesis regarding memory amplification (Southwick et al., 1997) or more source monitoring errors (Lindsay & Johnson, 2000) for disgust. Given memory amplification occurs when people remember more trauma-related details *over time* (Oulton et al., 2016), future research should examine whether disgust’s liberal response bias becomes more liberal over time, perhaps leading participants to say yes to more (old and new) images than they originally saw. Indeed, participants had a liberal response bias for disgust (i.e., typically responded ‘old’ to disgust images) and a conservative response bias for fear (i.e., typically responded ‘new’ to fear images). These results suggest participants used a more lenient criterion when judging disgust—relative to fear—images. Put differently, if participants were unsure of whether they had previously seen an image, they favored identifying disgust images as ‘old’ and fear images as ‘new’. Notably, participants only showed a liberal response when we calculated response bias using old disgust images and *related* lures; perhaps they used a less lenient criterion because they felt surer about whether they previously saw the unrelated image lures.

There is a theoretical rationale for participants’ liberal response bias for disgust (and conservative bias for fear). Remembering disgust is important because disgust stimuli are subtle, easily/quickly spread, and resistant to decay (Chapman et al., 2013). This ease-of-transmission occurs for contamination-related disgust (e.g., infectious diseases) and for moral disgust (e.g., viewing a person who associates with immoral people—like paedophiles—as disgusting; Giner-Sorolla et al., 2018). Consistent with this idea, participants were more inclined to judge a ‘new’ disgust image as ‘old’ (‘false alarm’) than judge an ‘old’ disgust image as ‘new’ (‘miss’). Given the (real or perceived) ‘permanency’ of disgust, people may judge disgust more liberally because the consequences of missing a disgust threat (e.g., contracting an illness, social rejection) outweighs the consequences of falsely remembering disgust. Participants’ conservative response bias for fear suggests they ‘missed’ fear images. Given the ‘fleeting’ (nonpermanent) nature of fear-inducing situations, people pay *less* attention to fear stimuli (Carretié et al., 2011). Thus, they may feel less confident in their memory, rejecting more (‘old’ and ‘new’) fear targets.

Our findings have clinical implications for understanding the interplay between disgust, memory, and PTSD. We found that false memories were not exacerbated for disgust images and thus are not a disgust-specific factor that may worsen PTS symptoms, for example. However, our finding that people had a stronger memory trace (i.e., better memory sensitivity) for disgust relative to fear may provide insight into why disgust is more difficult—compared with fear—to extinguish through exposure therapy (a commonly endorsed intervention for treating PTSD; Harned et al., 2015; Mitchell et al., 2024; Zeng et al., 2021). Exposure therapy relies on reversing classical and operant conditioning processes, wherein a person is exposed to their trauma-related memories in a gradual manner (Bryant, 2019). The goal of exposure therapy is to reduce the intensity of emotions associated with these trauma-related memories, and subsequent avoidance behaviors. However, disgust’s strong memory trace, the evaluative nature in which disgust is conditioned, and the risks associated with forgetting disgust (given disgust’s ‘permanency’), may explain why disgust memories are difficult to manipulate/reduce in PTSD treatments (Mitchell et al., 2024).

Our findings yield methodological considerations. Given we found higher false-memory rates—for *related* lures only—than prior studies (e.g., Chapman et al., 2013), future research should use old/related lure pairs normed on similarity. A limitation of our study is that—despite extensive piloting—the disgust and fear image sets were not equivalent on memory-enhancing variables. Disgust images were more negative (less pleasant, more unpleasant) than fear images, and fear images more arousing than disgust images. Furthermore, our disgust images elicited disgust (*M* = 5.0) to a greater extent than our fear images elicited fear (*M* = 4.6). However, these differences were small, seen in other studies (Chapman et al., 2013; Matson et al., 2024), and likely reflect real-world differences between disgust and fear (Faith & Thayer, 2001).

Disgust and fear are negative and arousing emotions with distinct cognitive effects. We found disgust memory enhancement extends to accurate memory, evidenced by fewer false memories overall, higher correct recognition rates, and better memory sensitivity for disgust than fear images. Thus, disgust is a particularly memorable emotion, which may in part explain why disgust memories are more resistant to extinction than fear memories in targeted interventions for PTSD.

## Supplementary information

Below is the link to the electronic supplementary material.

Supplementary file1 (DOCX 44 KB)

## Data Availability

We preregistered this study (https://osf.io/vbs9w); data (https://osf.io/7v6ax) and image codes (https://osf.io/3d67w) are publicly available.

## Footnotes

1 We did not closely match our disgust and fear image sets on interrelatedness (another memory-enhancing variable; Talmi et al., 2007) in the current study because memory recognition tests are relatively immune to differences in interrelatedness (Chapman et al., 2013).

2 At least 50 participants rated each image pair on similarity.

3 Descriptive statistics for image ratings per the three disgust and three fear image sets appear in Supplementary Table 2.

4 Descriptive and inferential statistics for the neutral images are reported in Supplementary Table 3.

5 Participants who did not closely attend to the images (i.e., reported ≤4 on the scale; *n* = 2) and/or who closed their eyes/looked away from >4 images (*n* = 4) were already excluded from the study (due to not following instructions for the LDT or not completing Part 2).

6 Five participants did not complete the recognition test within this time frame, and instead responded within 72 hours (following an additional reminder email at 48 hours post-encoding, as preregistered); the average response time of these five participants was 54.3 hours post-encoding.

7 Inferential statistics for the interaction between emotion (disgust, fear, neutral) on false memories for *unrelated* lures are reported in Supplementary Table 4.

8 As preregistered, we removed extreme outliers (>3 *SD* per participant) from the analysis.
