# Supplementary material for: Disgust memory enhancement extends to more accurate memory but not more false memories
Source: Mem Cognit. 2025 Jan 20;53(6):1754–66. doi: 10.3758/s13421-024-01681-x (PMC12402033; doi:10.3758/s13421-024-01681-x)
Supplement: Supplementary file 1 — Supplementary file1 (DOCX 44 KB) [file 13421_2024_1681_MOESM1_ESM.docx]

Table of Contents

[Supplementary Table 1: 2](#_Toc168341129)

[Thematic descriptions of the content depicted in the disgust and fear images across the three image sets. 2](#_Toc168341130)

[Supplementary Table 2 3](#_Toc168341131)

[Ratings (M (SD)) for each disgust and fear image set. 3](#_Toc168341132)

[Supplementary Table 3 4](#_Toc168341133)

[Descriptive (Ms and (SDs)) and inferential statistics (independent samples t-tests) for neutral images. 4](#_Toc168341134)

[Supplementary Table 4 5](#_Toc168341135)

[Inferential statistics for the interaction between emotion (disgust, fear, neutral) on false memories for unrelated lures. 5](#_Toc168341136)

[Supplementary Table 5 6](#_Toc168341137)

[Descriptive statistics (M, SD, range) for posttraumatic stress and trait disgust measures. 6](#_Toc168341138)

[Supplementary Table 6 7](#_Toc168341139)

[Correlations between correction recognition (i.e., hits), false memories (i.e., false alarms), memory sensitivity (i.e., d’) and response bias (i.e., c) for disgust images, with trait disgust and PTS symptom measures. 7](#_Toc168341140)

[Image Piloting Rating Scales 10](#_Toc168341141)

[Recognition Test Instructions 11](#_Toc168341142)

# **Supplementary Table 1:**

## *Thematic descriptions of the content depicted in the disgust and fear images across the three image sets.*

|  | **Disgust** | | **Fear** | |
| --- | --- | --- | --- | --- |
|  | Set 1 and Set 2 (Pairs) Themes | Set 3 (Unrelated) Themes | Set 1 and Set 2 (Pairs) Themes | Set 3 (Unrelated) Themes |
| 1. | Animal head (deceased) | Fresh meat at a market | Young boys armed with weapons | Growling bear |
| 2. | Moldy bread | Chicken missing feathers | Bomb | Home invasion |
| 3. | Cockroach | Deformed foot | Car on fire | Shark |
| 4. | Hair in drain | Bloody nose | Clown | Girl screaming |
| 5. | Bloodshot eye | Dirty toilet | Bushfire | Person wearing balaclava |
| 6. | Leeches | Tick | Gun pointed at a woman | Growling dog |
| 7. | Maggots | Vomit | Person holding a knife | Soldier pointing a gun |
| 8. | Roadkill animal | Cricket covered in ants | Written-off vehicle (post-collision) | Police chase |
| 9. | Waste | Mouse intestines | Hostage | Physical assault |
| 10. | Scab | Cigarettes | Scary face | Armed group of people surrounding a car |
| 11. | Surgery | Lions feeding on a deceased zebra | Snake | Domestic violence |
| 12. | Rotten teeth | Bloody finger | Spider | Person holding a knife against someone else’s throat |

# **Supplementary Table 2**

## *Ratings (M (SD)) for each disgust and fear image set.*

|  | Disgust | | | Fear | | |
| --- | --- | --- | --- | --- | --- | --- |
| Rating Type | Set 1 Pair | Set 2  Pair | Set 3 Unrelated | Set 1 Pair | Set 2  Pair | Set 3 Unrelated |
| Arousal | 3.6  (1.8) | 3.6  (1.7) | 3.6  (1.8) | 4.0 (1.8) | 4.0  (1.9) | 4.0  (1.8) |
| Pleasantness | 1.3  (0.7) | 1.3  (0.7) | 1.4  (0.8) | 1.4  (0.9) | 1.5  (0.9) | 1.6  (1.0) |
| Unpleasantness | 5.4  (1.4) | 5.4  (1.4) | 5.4  (1.5) | 5.0  (1.6) | 5.0  (1.7) | 5.0  (1.5) |
| Distinctiveness | 4.4  (1.6) | 4.4  (1.5) | 4.8  (1.6) | 4.8  (1.5) | 4.8  (1.5) | 4.5  (1.6) |
| Disgust | 5.0  (1.6) | 5.0  (1.6) | 5.0  (1.6) | 2.7  (1.7) | 2.8  (1.9) | 2.6  (1.7) |
| Fear | 2.9  (1.8) | 2.8  (1.8) | 2.9  (1.9) | 4.6  (1.8) | 4.6  (1.8) | 4.6  (1.8) |

# **Supplementary Table 3**

## *Descriptive (Ms and (SDs)) and inferential statistics (independent samples t-tests) for neutral images.*

| Outcome Variable | *M* (*SD*) | *Emotion comparison for t-test* | *df* | *t* | *p* | 95% CI | Cohen’s *d* |
| --- | --- | --- | --- | --- | --- | --- | --- |
| Arousal | 2.0 (0.5) | Neutral vs. Disgust | 58 | 8.82 | < .001 | 1.16 – 1.84 | 2.32 |
| Arousal |  | Neutral vs. Fear | 39.75 | 16.84 | < .001 | 1.72 – 2.19 | 4.70 |
| Pleasantness | 4.5 (0.8) | Neutral vs. Disgust | 24.92 | -19.46 | < .001 | -3.55 –  -2.87 | -6.15 |
| Pleasantness |  | Neutral vs. Fear | 27.36 | -17.79 | < .001 | -3.36 –  -2.66 | -5.47 |
| Unpleasantness | 1.3 (0.2) | Neutral vs. Disgust | 42.37 | 39.44 | < .001 | 3.86 – 4.28 | 8.71 |
| Unpleasantness |  | Neutral vs. Fear | 48.59 | 47.79 | < .001 | 3.50 – 3.81 | 10.82 |
| Distinctiveness | 2.8 (0.7) | Neutral vs. Disgust | 57 | 8.68 | < .001 | 1.32 – 2.11 | 2.32 |
| Distinctiveness |  | Neutral vs. Fear | 36.24 | 11.93 | < .001 | 1.61 – 2.28 | 3.43 |
| Disgust | 1.1 (0.1) | Neutral vs. Disgust | 38.07 | 38.26 | < .001 | 3.65 – 4.06 | 8.30 |
| Disgust |  | Neutral vs. Fear | 37.73 | 14.99 | < .001 | 1.38 – 1.82 | 3.25 |
| Fear | 1.2 (0.1) | Neutral vs. Disgust | 37.92 | 13.18 | < .001 | 1.45 – 1.97 | 2.86 |
| Fear |  | Neutral vs. Fear | 48.34 | 55.78 | < .001 | 3.33 – 3.58 | 12.61 |
| *Note.* *N* = 24 (except *N* = 23 for distinctiveness ratings) | | | | | | | |

# **Supplementary Table 4**

## *Inferential statistics for the interaction between emotion (disgust, fear, neutral) on false memories for unrelated lures.*

| Analysis | Inferential Statistic |
| --- | --- |
| One-way repeated measures ANOVA (emotion: disgust, fear, neutral) | *F*(2, 220) = 12.75, *p* < .001, η_p_ = .10. |
| Simple contrast between disgust and fear | *M*_diff_ = .09; 95% CI [.05, .12], *p* < .001; η_p_^2^ = .16 |
| Simple contrast between disgust and neutral | *M*_diff_ = .02; 95% CI [-.03, .07], *p* = .37;  η_p_^2^ = .007 |
| Simple contrast between fear and neutral | *M*_diff_ = .1; 95% CI [.06, .2], *p* < .001; η_p_^2^ = .15 |

Contrary to our prediction, when image lures depicted content that was unrelated to the content in images presented during encoding, participants experienced *more* false memories of fear compared to disgust and neutral images, and a *similar* proportion of false memories of disgust and neutral images. These results are somewhat inconsistent with findings from past research. For example, Schienle et al. (2021) and Chapman et al. (2013) found no difference between false memory rates for disgust, fear and neutral images, while Marchewka et al. (2016) found higher false memory rates for disgust and fear images, relative to neutral images. Given these mixed findings, we cannot draw robust conclusions about the differences between neutral, disgust and fear images. Nevertheless, across all studies there were never higher false memory rates for neutral relative to negative images. Thus, results from past research and the present study support emotionally enhanced memory, which posits that people accurately *and* falsely remember more emotional than neutral stimuli.

# **Supplementary Table 5**

## *Descriptive statistics (M, SD, range) for posttraumatic stress and trait disgust measures.*

| Questionnaire | *M* | *SD* | *Range (minimum – maximum)* |
| --- | --- | --- | --- |
| PCL-5 total | 15.1 | 17.5 | 0 – 65 |
| PCL-5 re-experiencing subscale | 3.6 | 4.7 | 0 – 17 |
| PCL-5 avoidance subscale | 2.4 | 2.6 | 0 – 8 |
| PCL-5 negative alterations in cognition and   mood subscale | 5.0 | 6.7 | 0 – 24 |
| PCL-5 alterations in arousal and reactivity   subscale | 4.2 | 5.2 | 0 – 20 |
| DPPS-R disgust propensity subscale | 16.5 | 4.2 | 6 – 28 |
| DPPS-R disgust sensitivity subscale | 12.1 | 5.0 | 6 – 26 |
| DAQ total | 85.2 | 21.4 | 17 – 119 |
| DAQ behavioral subscale | 45.6 | 11.3 | 9 – 63 |
| DAQ cognitive subscale | 39.6 | 11.2 | 8 – 56 |
| DAQ prevention subscale | 45.2 | 11.8 | 9 – 63 |
| *Note. N* = 111. PCL-5: Posttraumatic Stress Disorder Checklist; DPSS-R: Disgust Propensity and Sensitivity Scale – Revised; DAQ: Disgust Avoidance Questionnaire. On average, participants reported subthreshold PTSD symptom levels based on the PCL-5 cut-off (31; Ashbaugh et al., 2016). According to this cut-off, 19.8% of the sample (n = 22) were PTSD-probable. | | | |

# **Supplementary Table 6**

## *Correlations between correction recognition (i.e., hits), false memories (i.e., false alarms), memory sensitivity (i.e., d’) and response bias (i.e., c) for disgust images, with trait disgust and PTS symptom measures.*

|  | Hits | False alarms (related lures) | False alarms (unrelated lures) | *d*’ (hits vs. related lures) | *d*’ (hits vs. unrelated lures) | *c* (hits vs. related lures) | *c* (hits vs. unrelated lures) |
| --- | --- | --- | --- | --- | --- | --- | --- |
| DPSS-R disgust propensity subscale | .12  [-.07, .30] | -.03  [-.22, .16] | .07  [-.12, .25] | .11  [-.08, .30] | .05  [-.14, .24] | -.06  [-.24, .13] | -.13  [-.31, .06] |
| DPSS-R disgust sensitivity subscale | -.0004  [-.19, .19] | -.04  [-.23, .14] | .13  [-.06, .31] | .06  [-.13, .24] | -.08  [-.26, .11] | .02  [-.17, .20] | -.10  [-.29, .09] |
| DAQ behavioral subscale | .11  [-.08, .29] | -.09  [-.27, .10] | -.09  [-.27, .10] | .12  [-.07, .30] | .13  [-.06, .31] | .02  [-.17, .20] | .0005  [-.19, .19] |
| DAQ cognitive subscale | .06  [-.13, .25] | -.07  [-.25, .12] | -.20  [-.37, -.01] | .07  [-.12, .25] | .19  [.001, .36] | .008  [-.18, .20] | .11  [-.08, .29] |
| DAQ prevention subscale | .06  [-.13, .25] | -.07  [-.25, .12] | -.15  [-.33, .03] | .06  [-.13, .25] | .14  [-.05, .32] | .01  [-.17, .20] | .08  [-.11, .27] |
| DAQ total | .09  [-.10, .27] | -.08  [-.26, .11] | -.15  [-.33, .04] | .10  [-.09, .28] | .16  [-.02, .34] | .01  [-.18, .20] | .06  [-.13, .24] |
| PCL-5 re-experiencing subscale | .00007  [-.19, .19] | .19  [.004, .36] | .20  [.01, .37] | -.10  [-.28, .09] | -.08  [-.27, .11] | -.18  [-.36, .01] | -.17  [-.35, .02] |
| PCL-5 negative alterations in cognition and mood subscale | -.01  [-.20, .17] | -.04  [-.22, .15] | .09  [-.10, .27] | .06  [-.13, .24] | -.04  [-.23, .14] | .02  [-.17, .21] | -.07  [-.26, .12] |
| PCL-5 avoidance subscale | -.008  [-.19, .18] | .14  [-.05, .32] | .07  [-.12, .25] | -.07  [-.26, .12] | -.02  [-.21, .16] | -.09  [-.27, .10] | -.05  [-.24, .14] |
| PCL-5 alterations in arousal and reactivity subscale | -.06  [-.25, .13] | .005  [-.18, .19] | .09  [-.09, .28] | .002  [-.19, .19] | -.07  [-.25, .12] | .004  [-.18, .19] | -.05  [-.24, .14] |
| PCL-5 total | -.03  [-.21, .16] | .06  [-.13, .24] | .13  [-.06, .31] | .02  [-.20, .17] | -.06  [-.25, .13] | -.05  [-.24, .14] | -.10  [-.28, .09] |
| *Note.* *N* = 111 for hits and false alarm analyses; *n* = 109 for related lure *d*’ and *c* analyses; *n* = 110 for unrelated lure *d*’ and *c* analyses. Bonferroni adjusted for unrelated and related lures (*p* = .025 for all memory variables except for ‘Hits’). DPSS-R: Disgust Propensity and Sensitivity Scale – Revised; DAQ: Disgust Avoidance Questionnaire; PCL-5: Posttraumatic Stress Disorder Checklist. | | | | | | | |

# **Image Piloting Rating Scales**

How *pleasant* would you rate this image? (1 = not at all, 7 = extremely)
How *unpleasant* would you rate this image? (1 = not at all, 7 = extremely)
How *emotionally arousing* is this image? (1 = not at all, 7 = highly)

How *disgusting* is this image? (1 = not at all disgusting, 7 = extremely disgusting)
How *frightening* is this image? (1 = not at all frightening, 7 = extremely frightening)

How *distinctive* (unusual/eye-catching) would you rate this image? (1 = not at all distinctive, 7 = extremely distinctive)

How *similar* would you rate these two images? (1 = not very similar, 7 = very similar)

***Instructions for similarity ratings***

You will be shown pairs of photos, some of which are graphic and negative in nature. Please rate each pair of photos on their similarity (i.e., how close the resemblance is between the two images in the pair).  

# **Recognition Test Instructions**

Next, you will be shown a series of images one at a time. For each image, you will be asked if the photo is OLD or NEW.

Please select the OLD button if you saw the image yesterday in Session 1 of this study. Please select the NEW button if you did not see the image yesterday in Session 1 of this study.

Then, please rate your confidence in your decision for each image.

Sometimes you may be asked to identify if you REMEMBER or KNOW viewing that image.

Recognition memory often brings back to mind the context you first experienced. For example, you might recognize someone's face, and explicitly remember talking to this person at a party last week. At other times, you may recognize something and have a vague feeling that you have had prior exposure to it, but nothing comes to mind about the context. For example, someone’s face feels familiar, but you have no explicit recollection of seeing this person before.

For this study, please select REMEMBER if you recognize the image as one you saw yesterday in Session 1 of this study, and explicitly remember something you thought or experienced when the image appeared then. Alternatively, please select KNOW for each image that seems familiar, but you do not explicitly recall viewing it yesterday in Session 1 of this study.
